# Screening of the *LTBP2* gene in a north Indian population with primary congenital glaucoma

**Published:** 2013-01-17

**Authors:** Kuldeep Mohanty, Mukesh Tanwar, Rima Dada, Tanuj Dada

**Affiliations:** 1 Laboratory for Molecular Reproduction and Genetics, Department of Anatomy, All India Institute of Medical Sciences, New Delhi, India; 2 Dr. Rajendra Prasad Centre for Ophthalmic Sciences, All India Institute of Medical Sciences, New Delhi, India

## Abstract

**Purpose:**

Primary congenital glaucoma (PCG), a severe form of glaucoma that presents early in life, is an autosomal recessive eye disorder that results from defects in anterior eye segment. Null mutations in *LTBP2* were reported in patients with PCG in Pakistani and Iranian families. This study was aimed to identify the mutation profile of the *LTBP2* gene in north Indian patients with PCG.

**Methods:**

After ethical clearance, 54 unrelated patients with PCG who were either negative or heterozygous for *MYOC*, *CYP1B1*, and *FOXC1* mutations and 50 ethnically matched non-glaucomatous controls were recruited for the study. PCG diagnosis was established by the presence of buphthalmos in at least one affected eye and associated high intraocular pressure before the age of 3 years. *LTBP2* was screened in genomic blood DNA for mutations, with PCR and direct sequencing of PCR amplified fragments.

**Results:**

We observed one intronic single nucleotide polymorphism (rs3742793) between exons 6 and 7 in the *LTBP2* gene in 18 patients with PCG. This nucleotide change resulted in cytosine (C) being replaced by guanosine (G) at position g.75070493. No pathogenic variants were identified in the *LTBP2* gene in our cohort of patients.

**Conclusions:**

*LTBP2* gene mutations are not involved in the pathogenesis of primary congenital glaucoma in our patients. Thus, it is important to screen other glaucoma-associated loci and genes for involvement in congenital glaucoma in cases that are either negative or heterozygous for *MYOC*, *CYP1B1*, and *FOXC1* mutations to have better insight into the disease pathogenesis.

## Introduction

Glaucoma includes a set of ocular disorders involving optic nerve degeneration [1] and if is left untreated can cause permanent loss of vision [2]. The optic nerve damage involves degeneration of the retinal ganglion cells (RGCs) [3]. Impairment of RGCs results in visual field loss. Glaucoma is the second largest cause of blindness worldwide affecting approximately 65 million people [4]. Based on age of onset, etiology, and anatomy of the anterior chamber, glaucoma is broadly classified into three types [2]. They are closed angle glaucoma, primary open angle glaucoma, and primary congenital glaucoma (PCG). PCG (PCG: OMIM 231300) is a severe form of glaucoma that presents early in life; that is, the age of onset for PCG falls in the infantile (before the age of three) or neonatal period [5]. PCG is associated with developmental anomaly of the anterior eye segment. The main feature of PCG is the dysgenesis of trabecular meshwork cells that leads to hindrance in the aqueous outflow pathway. These anatomic changes lead to elevated intraocular pressure (IOP) and optic nerve damage [6]. Clinical features associated with this disorder include photophobia, epiphora, blepharospasm, elevated IOP, enlargement of the globe (buphthalmos), corneal enlargement and edema, rupture of Descemet’s membrane (presence of Haab’s striae), and optic nerve damage [7,8].

PCG is an autosomal recessive eye disorder observed in sporadic and familial cases. The prevalence of PCG varies according to the geographical location and ethnicity. The incidence is 1:10,000 in Western countries, 1 in 2,500 in Saudi Arabia, and highest in the Gypsy population of Slovakia, where the incidence is 1 in every 1,250 live births owing to the high rate of consanguinity in these countries [5,8]. The prevalence of PCG in southern India is approximated to be 1 in 3,300 and causes 4.2% of childhood blindness [9]. PCG is a heterogeneous genetic disease. Three loci have been identified: GLC3A (2p21) [10], GLC3B (1p36) [11], and GLC3C (14q24.3) [12]. Until recently, only two candidate genes viz. *CYP1B1* (OMIM *601771; cytochrome P450, subfamily I, polypeptide I) and *LTBP2* (OMIM *602091; latent transforming growth factor beta binding protein 2) have been reported for PCG [7] [13]. Mutations in *CYP1B1* (cytochrome P450, family 1, subfamily B) at the GLC3A locus explain only a proportion of the PCG alleles [5]. Mutations in the *CYP1B1* gene have been reported in Indian patients with PCG [14].

A study in consanguineous Pakistani families with PCG revealed linkage to a new locus adjacent to GLC3C on 14q24.2–24.3 [15]. *LTBP2* is the identified gene in this locus. Homozygous nonsense mutations in the *LTBP2* gene were identified in four consanguineous Pakistani families and in eight of 15 Gypsy individuals with primary congenital glaucoma. Patients with PCG from Pakistan and Iran were reported to harbor mutations in this gene while studies on patients with PCG from Saudi Arabia ruled out the presence of these or any other mutations in this gene [13,16,17]. These findings support the fact that incidence of mutations in the *LTBP2* gene in patients with PCG is not a universal observable fact. No studies have analyzed *LTBP2* in an Indian population with PCG. In the present study, we screened all the exons with intron-exon boundaries of the *LTBP2* gene in 54 unrelated patients with primary congenital glaucoma who were either negative or heterozygous for *CYP1B1*, *MYOC*, and *FOXC1* gene mutations and 50 healthy controls.

## Methods

### Clinical evaluation and patient selection

75 patients diagnosed with primary congenital glaucoma presenting at the Dr. Rajendra Prasad Centre for Ophthalmic Sciences, All India Institute of Medical Sciences (AIIMS), New Delhi, India, were enrolled in the study. Out of the 75 patients 51 were male and 24 were female. The age of onset of the disease ranged from birth to 1 year. This study was performed according to the tenets of the Declaration of Helsinki. After ethical approval was received from the Institutional Review Board (IRB00006862; AIIMS), a total of 54 patients with PCG were screened for *LTBP2* sequence variations (either negative heterozygous for *MYOC*, *CYP1B1* and *FOXC1* gene mutations; Appendix 1). All patients were unrelated to each other and were examined by one of the authors (glaucoma specialist). The diagnosis involved clinical, ocular, and systemic examination. Inclusion criteria for the patients were increased corneal diameter (>12.0 mm) and raised IOP (>21 mmHg) with the presence/absence of Haab's striae and optic disc changes (where examination was possible). The additional inclusion factors were symptoms of epiphora and photophobia.

All patients with other ocular anomalies or with systemic glaucoma associated syndrome and with history of blood transfusion, toxoplasmosis, rubella, cytomegalovirus, and herpes simplex virus (TORCH) infection, and drug intake by mother during pregnancy were excluded from the study. Detailed family history of ocular or other hereditary disorders up to three generations were taken, and pedigree charts were constructed.

### Control group

Fifty ethnically matched non-glaucomatous individuals without any ocular/systemic disorders were enrolled as controls. Controls were unrelated to patients and to each other. All were older than 18 years and had normal IOP, open angles on gonioscopy, and normal optic nerves on examination. Informed consent forms were signed, and peripheral blood samples were collected from patients and controls by venipuncture in ethylenediaminetetra-acetic acid (EDTA) vacutainers and stored in −80 °C until further use.

### Mutation screening and sequence analysis

Genomic DNA was extracted from whole blood samples using the organic method described by Sambrook et al. [18], with some modifications. Briefly, equal volume of lysis buffer was added to the blood samples and centrifuged. To the pellet, DNA extraction buffer, Proteinase K and sodium dodecyl sulfate (SDS) was added and incubated at 37 °C overnight. Equilibrated phenol and chloroform: isoamylalcohol was added to the suspension and mixed. The suspension was centrifuged at 5000 × *g* for 10 min. DNA was precipitated from the upper viscous layer using ice-cold ethanol. All 36 exons of *LTBP2* with exon-intron boundaries were amplified from genomic DNA using PCR. Twenty-nine sets of overlapping primers were designed using the National Center for Biotechnology Information (NCBI) PRIMER3 program (Table 1). PCR amplification was performed in a S1000 thermal cycler (Bio-Rad, Hercules, CA). Amplifications were done in a 25 μl volume containing 3.0 pM of each primer (Integrated DNA Technology, Coralville, IA), 100 ng of genomic DNA, 1 unit of Taq polymerase (Banglore Genei P Ltd, Bengaluru, Karnataka, India), 0.1 mM of each deoxyribonucleotide triphosphate (dNTP), and 3.0 µl of 10× PCR buffer (with 15 mM MgCl_2_), with 35 cycles of amplification, each consisting of 30 s denaturation at 94 °C, 60 s annealing ranging from 59 °C to 62 °C and 1 min extension at 72 °C, and final extension at 72 °C for 5 min.

**Table 1:** PCR primers used for amplification of *LTBP2* gene

S.No.	Forward Primer	Reverse Primer	Product size (bp)
1	GCCGACCACAAAGCTCTTC	CAGAGGGACGAGGGTATGA	663
2	GATGTGCAGAGAATGGCAGA	TCAAGTGATCCACCCACCTT	530
3	AGAGTGGCTTCCTGCTTGAG	CAGCCCCAACACCTACTCTC	580
4	CTCAGGGCACCTTCATGTCT	AACTCAGCCCCTCTGTGAGA	450
5	AATGCCCTTGAGATGAATGC	CTAGGCTGCCAAGTGAGGAC	443
6	CAGGAGCCATCTAGGGTCAG	CAGCTTCCCTATCCCTGTCA	415
7	TCAGAGGGTTGGAAATGAGG	AGAGGAGGAGAAGGGCAGAC	429
8	TGCTTCCTTCTGGGATATGG	GACAGACTGCACCAGCAGAG	475
9	AGGTGGGCTGAGAGGAGTCT	TCTCAAGCAAGTCCCTGGAT	440
10	GAAACTGAGGCACAGGGAGA	GCCCAACTCCAGGTTGAATA	430
11	GCTCCAAACTTCCCAACTGA	TGCTGGAAACTTAGGGGAAA	837
12	GACCTGGGGTTCTGGAATTT	TCCTCCCACTTGGTCATCTC	383
13	TGTGTAAAGTGCCTGGCAGA	AGCTCCCAGAAACAGCACTC	377
14	GTCTGAGCACCAGGGAAGAG	GAGGGACCCTGTGTTCTTTG	370
15	GGTCCCCTAGGGTCTTATGC	TGCTTGGACCTTCTGCTTCT	408
16	TGGGCTGACTTTATGGCTTC	GGATTTCTACCCCTCCTTGC	510
17	ATCCTTTGTCCTTGGCCTCT	AGAAGGCTGACACTCCCAGA	567
18	AACAGCCCAGCTCACAAAGT	ACCTCTTTCCCTTTCCGTGT	504
19	CCCTGGCCTCATAACTGAGA	CCAAACTGGGGACAAATTGA	659
20	ACGGTGAGGTTCCTGCATT	CTGGCTTCCCATGCTCCT	883
21	GCCCAGAGGAAGCTACACAG	TTTACACGAAGCCTTCAGCA	566
22	AGAACCCCAGAGGTTGTGG	CAGGACCAGTTGAGGAGGAG	782
23	TTGGAGAATGTGCACTGAGG	CCTGTAGCTCCTGGTTTTGC	597
24	GGCCACTTCTTAGGGTTGTG	CTGGGACAGAAAAGGTGGAG	695
25	GCAAGGCGAACTTAAGCAAC	GGAAGGGTGTTTGCCTATCA	534
26	GTCAGAGATTGTCCCCAGGA	ACTTTGTCCCCAAACAGCAG	691
27	AGAGGTGGGGAGAGGAATGT	GGTGGAGGAGATGGAAGTGA	482
28	TCCCAGCATTAGGGAGAGTC	TTCCCAAAACCAAGCAACTC	494
29	GCTTGGTTTTGGGAAGTGAC	CCAAATCCTTTCTTGCTCCA	757

All PCR products were analyzed on 1.8% agarose gel, stained with ethidium-bromide (EtBr 10 mg/ml). Agarose gel was analyzed using a gel documentation system (Applied Biosystems, Carlsbad, CA). Successfully amplified PCR products were purified using a gel/PCR DNA fragments extraction kit (Catalog number DF100; Geneaid Biotech Ltd., Sijhih City, Taiwan). Purified PCR products were sent for sequencing to MCLAB (Molecular Cloning Laboratories, South San Francisco, CA). DNA sequences were analyzed against the *LTBP2* reference sequence (ENSG00000119681) using ClustalW2, a multiple sequence alignment tool for DNA provided by the European Molecular Biology Laboratory (EMBL) European Bioinformatics Institute (EBI). The effect of the sequence alterations on splicing was determined using NNsplice 0.9.

## Results

Clinical information for all patients with PCG is presented in Appendix 1. total of 104 participants including 54 cases and 50 age-matched controls were enrolled in this study. No consanguinity was found in any of the cases. All cases were sporadic with no family history. Among the 54 probands, 38 (70.30%) were male, and 16 (29.70%) were female. The age of onset ranged from birth to 1 year. The mean age of case presentation was 20 months, and the minimum age was 1 month to a maximum of 132 months. Buphthalmos was seen in 94% patients and 82% had bilateral and 18% had unilateral buphthalmous. Haab’s striae was present in only five cases (9%) and absent in 49 cases (91%). Corneal edema was present in 19 cases (35%) and absent in the remaining 35 (65%) cases. The mean corneal diameter, measured under general anesthesia, was 13 mm. The mean measured IOP before the first surgical procedure was 23.3±5.6/24.5±5.4 mmHg (OS/OD). The mean cup disc ratio (OS/OD) was 0.5:1/0.6:1.

**Figure 1:**
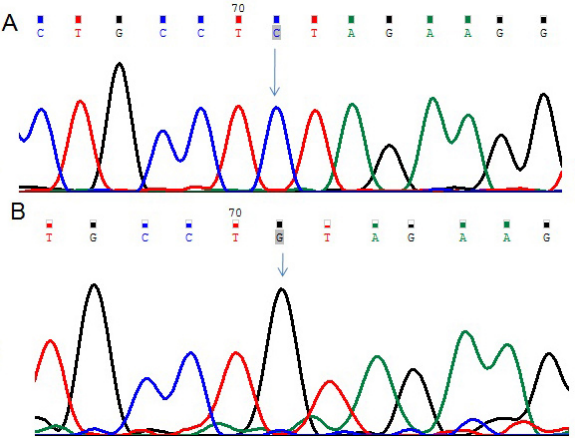
DNA sequence from exons 6 and 7 of *LTBP2*. **A**: The reference sequence derived from the control is shown. **B**: The sequence derived from the patient with congenital glaucoma shows the homozygous C>G (g.75070493C>T nucleotide change, a polymorphism (rs3742793) that has already been reported.

The 36 exons of the *LTBP2* gene were screened in 54 patients with PCG and 50 non-glaucomatous controls. A single nucleotide change (rs3742793) between exons 6 and 7 of the *LTBP2* gene was observed. This nucleotide change resulted in replacement of cytosine (C) by guanosine (G) at position g.75070493 (Figure 1). Eighteen of the patients with PCG carried this nucleotide change. The effect of this nucleotide change on splicing was analyzed and had no impact on the splicing of the RNA. None of the controls showed the presence of this nucleotide change in the *LTBP2* gene.

## Discussion

In this study, we screened north Indian patients with PCG for the *LTBP2* gene who were negative or heterozygous for *CYP1B1*, *MYOC*, and *FOXC1* gene mutations in one of our previous studies [14,19]. The latent tumor growth factor-β-binding proteins (LTBPs) are a superfamily of large, multidomain proteins with structural and tumor growth factor-β-signaling roles in the extracellular matrix. *LTBP2*, a member of this superfamily, is located on chromosome 14 and codes a matrix protein of 1,821 amino acids. *LTBP2* expression has been shown in human eyes, including the trabecular meshwork and ciliary processes reported to be relevant to the pathophysiology of primary congenital glaucoma and thus found to be associated with glaucoma [17]. *LTBP2* is the second gene implicated in PCG to date, but the precise mechanism by which mutations in this gene lead to PCG is still not clearly understood. After screening all 36 exons of the *LTBP2* gene, we identified one intronic single nucleotide polymorphism (rs3742793) between exons 6 and 7 in patients with PCG. Truncating and null mutations in *LTBP2*, encoding latent transforming growth factor-β-binding protein-2, were recently reported in patients with PCG from Pakistan and Iran [13,17].

Although the *LTBP2* function is still unknown, the presence of *LTBP2* in elastic tissue and association with fibrillin containing microfibrils has been shown [20]. The role of *LTBP2* in tissue repair processes and in cell adhesion has also been suggested by various studies [21-23]. It has also been reported that the carboxyl and N-terminal of LTBP2 interacts with fibrillin 1 and fibulin-5, respectively [24,25], indicating that *LTBP2* has functions related to those of microfibrils and elastin fibers. Single-nucleotide polymorphisms in the *LTBP2* gene are reported to be associated with various pathological conditions such as Marfan’s syndrome [26], mineral density variation and fracture risk [27], and skeletal and cardiovascular abnormalities [28].

A total of 1,267 nucleotide variations of different types and in various diseases have been reported in the *LTBP2* gene (Table 2). Out of the 1,267 variations, 344 were non-synonymous, which resulted in an amino acid change in the encoded peptide sequence. Only some of the *LTBP2* mutations have been found to be associated with PCG (Table 3). Recently, homozygous mutations in *LTBP2* were reported in a syndrome of megalocornea, microspherophakia (small spherical lens), lens dislocation, and secondary glaucoma developing after age 3 years [29] and in isolated microspherophakia/lens dislocation [30]. Another recent study documented the absence of mutations in the *LTBP2* gene in 54 Saudi Arabian families with PCG [31]. Apart from these studies, no other studies have documented mutations in *LTBP2* in patients with PCG. In our cohort of patients, *LTBP2* gene mutations are not involved in the pathogenesis of PCG. Negative results for *LTBP2* mutations in different PCG populations strongly suggests the involvement of other loci and genes in the pathogenesis of patients with PCG who are negative for *CYP1B1*, *MYOC*, and *FOXC1* mutations. For a better understanding of the pathogenesis and genetic mechanism of congenital glaucoma, more studies involving the *LTBP2* gene in sporadic cases and families with PCG should be conducted. In addition, the discovery of new loci and candidate genes would further the genetics of this blinding optic neuropathy opening new avenues for PCG management and control. Strong genetic markers established from candidate gene studies and family studies would prove highly valuable for predicting the likelihood and severity of PCG.

**Table 2:** Summary of variations reported for *LTBP2* gene (Ensembl)

Number of variants	Type of variations	Description of variations
1267	ALL	All variations
4	Essential splice site	In the first 2 or the last 2 base pairs of an intron
9	Stop gained	In coding sequence, resulting in the gain of a stop codon
14	Frameshift coding	In coding sequence, resulting in a frameshift
344	Non-synonymous coding	In coding sequence and results in an amino acid change in the encoded peptide sequence
32	Splice site	1–3 bps into an exon or 3–8 bps into an intron
251	Synonymous coding	In coding sequence, not resulting in an amino acid change (silent mutation)
2	Coding unknown	In coding sequence within determinate effect
13	5 prime UTR	In 5 prime untranslated region
27	3 prime UTR	In 3 prime untranslated region
555	Intronic	In intron
332	NMD transcript	Located within a transcript predicted to undergo nonsense-mediated decay
99	Within non-coding gene	Located within a gene that does not code for a protein
2	Upstream	Within 5 kb upstream of the 5 prime end of a transcript
11	Downstream	Within 5 kb downstream of the 3 prime end of a transcript

**Table 3:** LTBP2 mutations till date

S. No.	Nucleotide Change	Amino acid change	Exon	Ethnicity	Ref/refSNP	Phenotype
1	G→A	pGln111X	Exon1	Pakistani	[12]; rs121918356	PCG
2	C→T	p.R299X	Exon4	Gypsy	[12]; rs121918355	PCG
3	dupC	p.Val600GlyfsX2	Exon9	Moroccan	[27]	Secondary Glaucoma
4	delG	p.A138PfsX278	Exon1	Pakistani	[12]	PCG
5	delC	p.Tyr1793fsX55	Exon36	Iranian	[15]	PCG
6	delC	p.Ser472fsX3	Exon7	Iranian	[15]	PCG
7	G→A	p.Leu429Leu	Exon 6	Iranian	[15]; rs61738025	PCG
8	c.5446dupC	p.H1816PfsX28	Exon36	South Indian	[28]	Microspherophakia
9	c.1012delT	p.S338fsX4	Exon 4	Saudi	[29]	Secondary Glaucoma
10	C→G	p.Pro989Arg	Exon19	Iranian	[15]; rs76172717	PCG
11	G→A	p.Arg1603His	Exon33	Iranian	[15]; rs75200417	PCG
12	c.4855C>T	p.Q1619X	Exon33	Saudi	[29]	Secondary Glaucoma
13	c.4313G>A	p.C1438Y	Exon29	Saudi	[29]	Secondary Glaucoma
14	C→G	g.75070493	Intronic (6–7)	North Indian	Current study (rs3742793)	PCG

## Appendix 1. Clinical phenotype of PCG cases.

To access the data, click or select the words “Appendix 1.” This will initiate the download of a compressed (pdf) archive that contains the file. Key: M- male; F- female; H- homozygous; h-heterozygous; X- times; Trab/Trab+MMC- combined trabeculotomy trabeculectomy and mitomycin C treatmen right eye; OS- left eye; OD-right eye; OU- both eyes; NA- not available.
